# The role of head and neck squamous cell carcinoma cancer stem cells in tumorigenesis, metastasis, and treatment failure

**DOI:** 10.3389/fendo.2012.00090

**Published:** 2012-08-03

**Authors:** Steven B. Chinn, Owen A. Darr, R. D. Peters, Mark E. Prince

**Affiliations:** Department of Otolaryngology – Head and Neck Surgery, University of Michigan,Ann Arbor, MI, USA

**Keywords:** head and neck squamous cell carcinoma, cancer stem cells, tumorigenesis, metastasis, CD44, ALDH

## Abstract

Head and neck squamous cell carcinoma (HNSCC) is the sixth most common cancer worldwide. Despite advances in diagnostic and therapeutic methods, survival of HNSCC remains unchanged over the last 30 years with treatment failure and metastases being the strongest indicators of poor outcome. Cancer stem cells (CSC) have been identified in multiple other solid tumors, including breast, prostate, and pancreatic carcinoma. Recently, a subpopulation of tumor cells has been identified in HNSCC based on the overexpression of the cellular marker CD44 and increased activity of aldehyde dehydrogenase. These cells have been designated CSC based on their stem cell-like properties: self-renewal, tumorigenesis, and the ability to recapitulate a heterogeneous tumor. Recent work looking at the role of HNSCC CSC in tumorigenesis has shown that CSC have a greater capacity for tumor growth, increased motility, and invasive characteristics; *in vivo* experiments confirm greater metastatic potential in CSC compared to non-CSC. Clinically, CSC enrichment has been shown to be enhanced in recurrent disease, treatment failure, and metastasis. CSC represent a novel target of study given their slow growth and innate mechanisms conferring treatment resistance. Further understanding of their unique phenotype may reveal potential molecular targets to improve therapeutic and survival outcomes in patients with HNSCC.

## INTRODUCTION

Worldwide, head and neck squamous cell carcinoma (HNSCC) is the sixth most common cancer, affecting over 400,000 patients, and leading to over 200,000 deaths annually. Over the last 30 years, there has been limited improvement in survival despite advancement in surgery, radiotherapy, and chemotherapy (Jemal et al., 2011; Siegel et al., 2011). Metastases and treatment failures account for the majority of deaths in HNSCC. Improved understanding of the mechanisms of HNSCC tumorigenesis, metastases and treatment failure may have a significant impact on the morbidity and mortality of HNSCC. It is imperative that we strive for a better understanding of HNSCC at the cellular and molecular level so that we might develop more efficient therapies that target the key pathways of disease initiation and progression.

**FIGURE 1:**
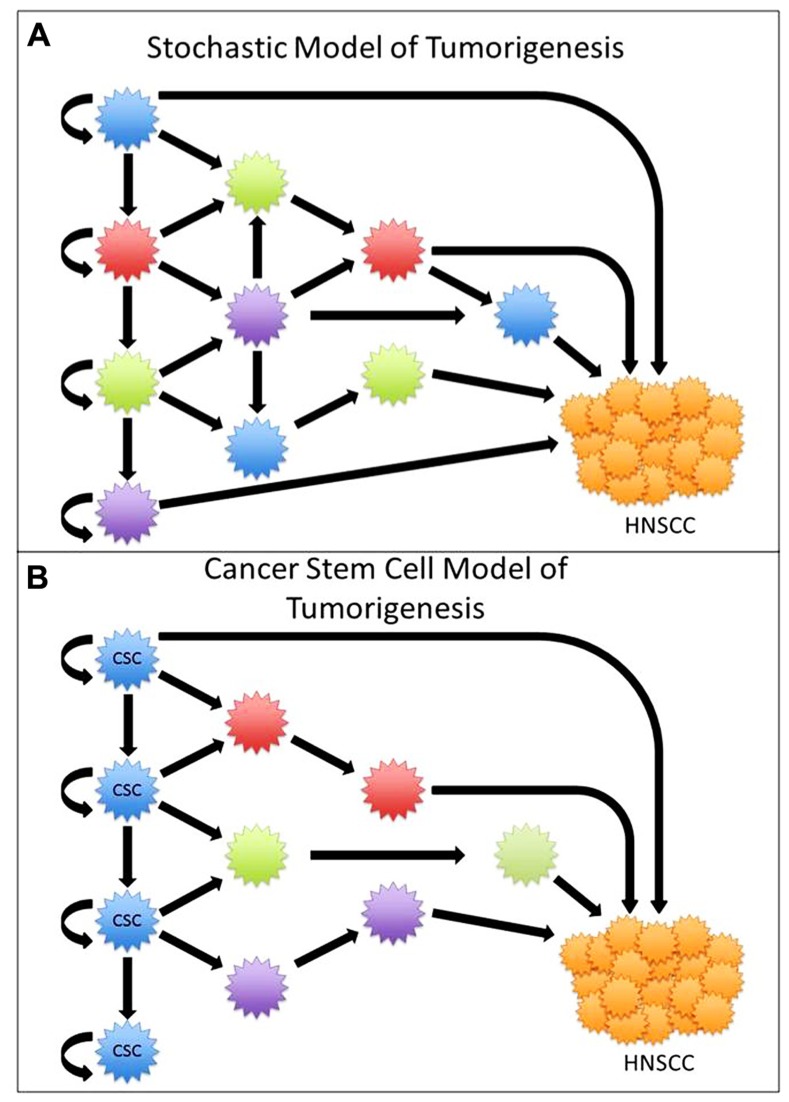
**(A)** In the stochastic model of tumorigenesis all cells are capable of renewal and tumorigenesis. **(B)** In the cancer stem cell (CSC) model, only a select group of cells are capable of asymmetric division and tumorigenesis.

Tumors were initially thought to arise from multiple different mutations within cells. As the number of mutations accumulates, cells ultimately become immortalized, where each cell is capable of initiating tumor growth (**Figure 1A**). The cancer stem cell (CSC) theory of tumorigenesis was originally proposed in the late 1970s, and has recently gained renewed interest due to identification of CSC in solid organ malignancies (Hamburger and Salmon, 1977; Vermeulen et al., 2008). The CSC theory is based on our understanding of embryological development and stem cell-derived organogenesis, where a few specific cells are capable of asymmetric division leading to the generation of diverse progenitor cells responsible for the creation of complex and heterogeneous organs. Similarly, the CSC theory explains that there exists a hierarchy of cells, where CSC are capable of unregulated asymmetric division, which is responsible for self-renewal and generation of a diverse population of differentiated progenitor cells that ultimately make up a heterogeneous tumor. According to the CSC theory, this subgroup of cells is responsible for the initiation of tumor growth and spread, whereas non-CSC are incapable of regenerating progeny or recapitulating a tumor (Reya et al., 2001; **Figure 1B**).

Subpopulations of highly tumorigenic cells, or CSC, were first described in hematologic malignancies in 1994 (Lapidot et al., 1994). Since then they have been identified in multiple solid organ malignancies using a variety of cellular markers. Al-Hajj and Clarke were the first to demonstrate CSC in breast cancer using the cellular markers CD44^+^/CD24^–/low^/Lineage^–^ cells. These cells are capable of serial regeneration, recapitulation of the primary tumor, and self-renewal of the tumor initiating cells (Al-Hajj et al., 2003; Wicha et al., 2006; Shah et al., 2007). Since then, CSC have been identified in multiple other solid organ malignancies, including CNS, pancreatic, lung, colon, and recently HNSCC (Singh et al., 2003; Kim et al., 2005; Li et al., 2007; O’Brien et al., 2007; Prince et al., 2007).

Work by Prince and colleagues in 2007 was the first to identify a subpopulation of HNSCC tumor cells with stem cell-like phenotypes, initially using the cellular marker CD44 (Prince et al., 2007). Like other CSC, these cells possess the stem cell-like qualities of self-renewal, tumorigenesis, and the ability to recapitulate a heterogeneous tumor. This marked the first identification of CSC in HNSCC. The purposes of this review are (i) to discuss the methods of CSC identification in HNSCC, (ii) to evaluate the HNSCC CSC as a mediator of tumorigenesis, metastasis, and treatment failures, and (iii) to evaluate the evidence for CSC as a potential target of therapy.

## CSC MARKERS

Cancer stem cells are often identified by specific molecular markers or enzymatic reactions. These CSC-specific markers allow for the use of flow cytometry to identify and sort a select subpopulation of cancer cells. The cellular makers CD44, CD133, and aldehyde dehydrogenase (ALDH) are used to identify head and neck CSC by fluorescence activated flow cytometry.

### CD44 AND CD133

CD44 is a cell surface glycoprotein that mediates cell–cell interactions by binding to hyaluronic acid and other extracellular ligands. Post-transcriptional and post-translational modifications to CD44 result in a diverse functional repertoire that includes aiding the adherence of leukocytes to endothelial cells and the invasion and subsequent metastasis of cancer cells (Wang et al., 2009). CSC can be isolated from HNSCC by fluorescence activated cell sorting (FACS) flow cytometry to identify cells stained with fluorescence-conjugated CD44 antibodies. CD44^high^ cells are characterized as having a more primitive, poorly differentiated morphology, and increased expression of known stem cell markers, such as BMI-1 (Prince et al., 2007, Song et al., 2006).

Similar to CD44, CD133 is a transmembrane glycoprotein characterized by its tendency to localize to cellular protrusions. CD133 is a protein commonly expressed in hematopoietic stem cells, endothelial progenitor cells, and various normal tissue stem cells. CD133 was first described as a CSC marker in leukemia and glioblastoma. Recently, it has also been described as a CSC marker in laryngeal squamous cell carcinoma as evidenced by cells with high CD133 expression exhibiting a CSC phenotype (Bonnet and Dick, 1997; Zhou et al., 2007). Like CD44, CD133-expressing cells can be identified using FACS.

### ALDEHYDE DEHYDROGENASE

Aldehyde dehydrogenase is a ubiquitous intracellular enzyme, catalyzing the oxidation of aldehydes in both physiologic and pathologic cellular processes. Two specific isoforms from the ALDH superfamily of proteins, ALDH1A1 and ALDH3A1, have been characterized for their pivotal role in both embryonic and adult stem cell physiology. ALDH was initially identified as a marker for embryonic and physiologic stem cells. More recently, ALDH has been shown to be a marker for CSC in hematologic and solid organ malignancies (Hess et al., 2004; Matsui et al., 2004; Brabletz et al., 2005; Pearce et al., 2005; Ginestier et al., 2007; Clay et al., 2010). Recently, expression of the ALDH1 isoform has been recognized as a marker for HNSCC and colon cancers. Unlike CD44 and CD133, the identification of tumor cells that express high levels of ALDH requires the use of the non-immunologic enzymatic ALDEFLUOR^TM^ Kit and FACS (Chen et al., 2009; Huang et al., 2009; Clay et al., 2010).

### SIDE POPULATIONS

The side population (SP) assay is another method of identifying stem cell-like populations. Goodell and colleagues isolated a SP of cells from bone marrow based on their ability to efflux the fluorescent DNA binding dye Hoechst 33342 compared to non-SP cells. SP cells are thought to be a source of CSC in hematologic and solid organ malignancies (Goodell et al., 1997; Hirschmann-Jax et al., 2004; Kondo et al., 2004; Ho et al., 2007; Wang et al., 2007; Burkert et al., 2008). Zhang et al. (2009) isolated SP cells from oral cavity tumors and found they had similar gene expression profiles to other CSC populations including overexpression of BMI-1 and OCT-4.

## TUMORIGENESIS

Cancer is defined by unregulated cell division and growth. CSC are believed to represent a mechanism for tumorigenesis and potentially offer a novel area of study for developing more effective treatments for HNSCC. HNSCC CSC were first described by Prince and colleagues in 2007 based on CD44 expression. In their experiments, they demonstrated enhanced tumorigenicity in the CD44^high^ subpopulation (as few as 5,000 cells) as compared to CD44^low^ cells, even when injecting >1 × 10^6^ cells (Prince et al., 2007). The resulting tumors, derived from the CD44^high^ injections, demonstrated renewal of CD44^high^ cells, indicating self-renewal, and regeneration of a heterogeneous tumor, thus meeting the definition of a CSC. Similar experiments using the enzymatic marker ALDH were also able to demonstrate that cells with ALDH^+^ expression had greater rates of tumorigenesis in mouse flank and neck injections (Chen et al., 2009; Clay et al., 2010). This work has been subsequently repeated by several other studies (Davis et al., 2010; Campos et al., 2012).

Gene expression signatures in ALDH/CD44-sorted HNSCC cells demonstrated BMI-1, a known CSC marker, to be differentially overexpressed, and when knocked down, demonstrated reduced tumorigenesis (Krishnamurthy et al., 2010). Based on these studies, cells expressing CD44^high^, ALDH^+^, and CD44^high^/ALDH^+^ demonstrate highly tumorigenic potential compared to their negative counterparts, while maintaining the CSC phenotype in perpetuity. However, CD44 and ALDH are not the only biomarkers capable of distinguishing cells capable of self-renewal. Sun and Wang (2011) looked at CMET^+^ cells compared to CMET^-^ and found increased tumorigenicity in a flank injections and found higher percentage of implantation in CMET^+^ cells compared to CD44^+^ cells and slightly lower than ALDH^+^ cells. In addition, Zhang et al. (2009) looked at HNSCC cell lines and oral cavity primary tumors identified the presence of SP cells. *In vitro* and *in vivo* analysis demonstrated SP cells had greater clonal expansion and greater tumorigenicity relative to non-SP cells. Although CD44 and ALDH are the most studied CSC markers, recent evidence supports the possibility that CSC, much like all tumor cells are heterogeneous in their genetic and expression signatures resulting in different phenotypes and varied capacities for tumorigenesis.

## METASTASIS

Regional and distant metastases in HNSCC correspond to an extremely poor prognosis with limited treatment options. Improved understanding of the mechanisms and etiology of metastases may allow for improvement in outcomes for patients with HNSCC. CSC have been linked with distant metastasis in breast cancer and pancreatic carcinoma. Analysis of bone marrow metastases has shown enrichment of cells expressing the breast CSC marker phenotype (CD44^+^/CD24^–^; Balic et al., 2006). In pancreatic adenocarcinoma a subgroup of pancreatic CSC expressing CD133^+^/CXCR4^+^ were shown to have an enhanced metastatic phenotype (Hermann et al., 2007).

In HNSCC, understanding the cellular mechanisms of invasion and metastasis is critical to developing new diagnostics and therapeutic modalities. CSC offer a unique mechanism for metastasis given their ability for tumor growth at the primary site, but also at the distant sites. *In vitro* and *in vivo* work has shown that HNSCC CD44^high^ cells have greater migration, invasion and metastatic potential compared to CD44^low^ cells (Davis et al., 2010). Gene expression studies comparing ALDH^+^ cells and ALDH^2013;^ cells demonstrated elevated levels of the metastatic and epithelial–mesenchymal transition (EMT) biomarkers CMET, TWIST, and SNAIL (Chen et al., 2011; Sun and Wang, 2011).

Side population cells have also been associated with metastasis. In two separate studies, SP cells were found to have higher incidence of metastasis in an intracardiac injection mouse model relative to non-SP and were highly enriched in metastatic lesions (Zhang et al., 2009; Song et al., 2010). These are important findings further isolating the genetic and expressome signatures in cells thought to initiate and propagate metastasis.

Collectively, these findings support CSC as important mediator and potential target in HNSCC metastasis. However despite these associations, the evidence and mechanisms of CSC mediated metastasis remains scant. Similar to tumorigenesis, CSC heterogeneity may also have an impact in a CSC ability to invade locally and metastasize distantly. Understanding the exact mechanisms remains elusive.

## TREATMENT FAILURES AND RESISTANCE TO THERAPY

Similar to the development of metastasis, treatment failure and recurrence portends a poor prognosis in HNSCC. Despite an increasing amount of research investigating the mechanisms responsible for treatment failure and resistance in HNSCC, outcomes remain largely unchanged. CSC have been shown to be especially resilient to toxic insult in a variety of malignancies, and may represent critical mediators of chemo- and radio-resistance within the diverse cellular population of a tumor. CSC possess unique mechanisms to resist cell death, including modified anti-apoptotic machinery, increased pump activity, and decreased cell division (Clarke et al., 2006). Glioblastoma and colorectal cells displaying CSC markers were enriched in the residual tumor population following treatment failures with standard chemotherapeutic agents (Kang and Kang, 2007; Dylla et al., 2008). When pancreatic carcinoma cells were incubated with gemcitabine, the proportion of CSC was significantly increased and cells with CSC markers exhibited more aggressive behavior (Shah et al., 2007). In addition to chemo-resistance, the CSC subpopulation in cervical cancer cells has been shown to resist radiation damage, and overexpresses genes related to radiation-resistance, DNA repair, hypoxia, and an invasive phenotype (Lopez et al., 2012).

In HNSCC, a higher percentage of CD44^+^ cells in a patient’s primary tumor has been shown to be associated with higher rates of treatment failure, while cells expressing the putative CSC markers CD44, CD24, Oct4, and integrin-b1 were associated with poor outcomes following radiotherapy (Joshua et al., 2012; Koukourakis et al., 2012). CSC, as defined by CD44 expression, have a greater resistance to pro-apoptotic stimuli (TNF-α and anti-Fas antibody) and a greater capacity for resistance to chemotherapeutic agents compared to non-CSC. At the molecular level, support for CSC-associated treatment resistance involves overexpression of anti-apoptotic genes and the multidrug resistant ABC transporters (Okamoto et al., 2009; Chikamatsu et al., 2012). In laryngeal SCC, cells overexpressing CD133 and ABCG2 demonstrated a significantly reduced cell death rate when co-cultured with common HNSCC-specific chemotherapy agents (Yang et al., 2011). Using ALDH as a marker of CSC, ALDH^+^ cells showed similar chemo- and radiation-resistant patterns, and interestingly, inhibition of the proposed CSC-mediator, SNAIL, caused reduced ALDH expression, decreased tumorigenesis and improved chemo- and radiation sensitization (Chen et al., 2009). With regard to SP cells, Zhang et al. (2009) demonstrated they possess qualities necessary for chemo-resistance, with elevated expression of ABC transporter proteins.

Invasive and metastatic behavior of epithelial cancers involves the loss of E-cadherin and transformation to a mesenchymal phenotype – a process known as epithelial–mesenchymal transition (EMT). Once cancer cells transition to a mesenchymal phenotype, they become increasingly motile and resistant to therapeutic options. Interestingly, Mani et al. (2008) established a relationship between these transformed cells and CSC; after induction of EMT in breast cancer cells, via activation of Snail/Twist, cells adopted stem-like properties of growth and tumorigenicity. In addition, just as hypoxia maintains the pluripotency of embryonic stem cells, a similar process may be involved with promotion of the CSC phenotype and its anti-apoptotic characteristics; hypoxia often represents low blood flow, which limits the distribution of chemotherapeutic drugs, and causes increased resistance to radiation, which requires sufficient oxygen tension to produce oxygen free radicals for cytotoxicity. Hypoxia inducible factors (HIFs) are overexpressed in CSC and may be responsible for some aspect of radiation-resistance in HNSCC (Vlashi et al., 2009). The enhanced mechanisms of CSC to endure and adapt to toxic insults may help explain treatment failures and poor outcomes in HNSCC, and a more sophisticated understanding of their unique survival machinery may illuminate points of vulnerability and lead to novel CSC-specific targets.

## CLINICAL IMPLICATIONS OF HNSCC CSC AND FUTURE AIMS

The use of CSC as markers for novel diagnostic and therapeutic targets is appealing. CD44 levels have been shown to be elevated in peripheral blood samples of HNSCC patients compared to healthy controls (Faber et al., 2011). Joshua et al. (2012) found that HNSCC primary tumors with elevated CD44^+^ cells (>36%) had an increased risk of recurrence of their primary tumor compared to tumors with lower CD44^+^ levels (<15%). The use of CSC markers for screening and early diagnosis may have a positive impact on outcomes. Likewise, understanding the enrichment of CSC in primary tumors may enhance our understanding of tumor behavior, enhance surveillance, and better predict outcomes.

**FIGURE 2:**
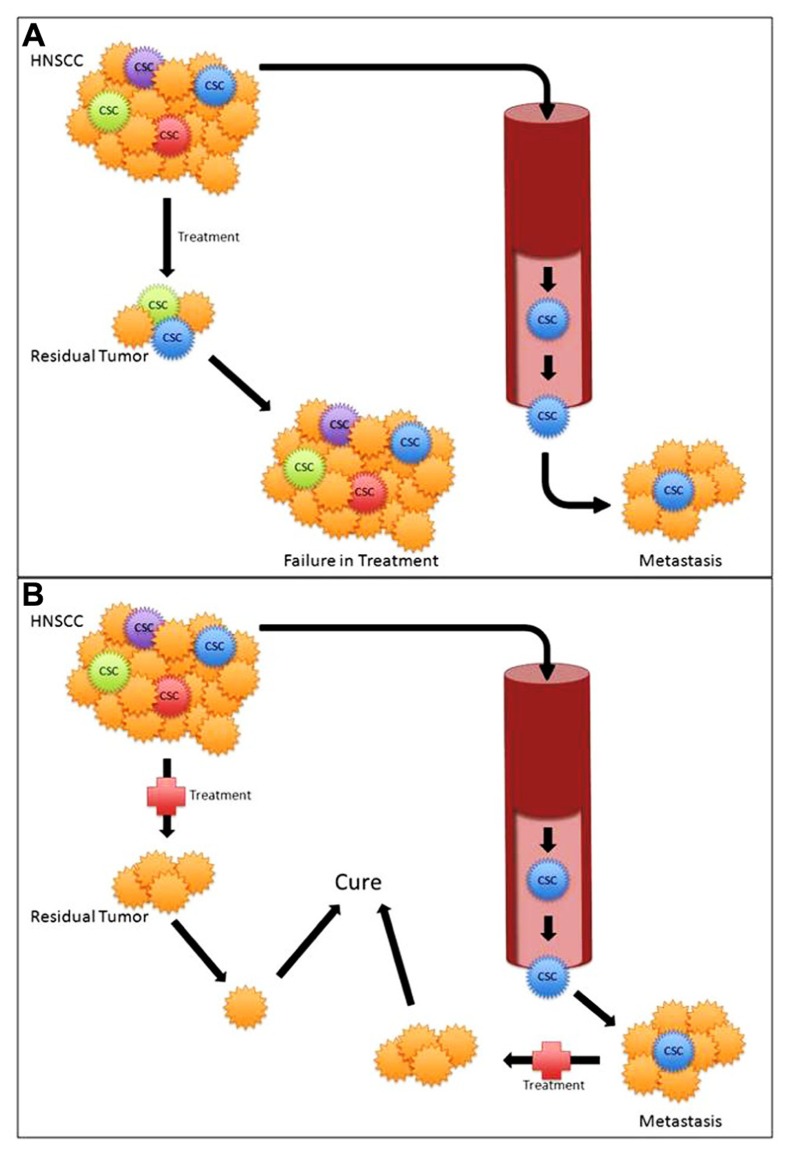
**(A)** Standard treatment modalities with cancer stem cell (CSC) mediated treatment failure and metastasis. **(B)** Targeted CSC therapy potentially leading to improved outcomes.

Increasing evidence supports CSC as mediators of tumorigenesis, metastasis, and treatment failure. Current therapies toward HNSCC are not CSC-specific and may result in inadequate CSC death. Given the evidence that only a small number of CSC are capable of tumorigenesis, any remaining CSC increase the risk of recurrence, metastasis, and ultimately poor outcomes (**Figure 2A**). CSC-directed treatment in HNSCC tumors is crucial in targeting the specific subpopulation of cells that is responsible for treatment failures and poor outcomes (**Figure 2B**). However, much like cancer biology in general, CSC biology is a complex heterogeneous process (Vermeulen et al., 2012). Similarly, not all CSC may have the same potential. Further study of HNSCC CSC heterogeneity and identifying CSC subsets may aid in better explaining CSC pathophysiology.

## CONCLUSION

The findings that head and neck CSC are intrinsic to the malignant physiology of tumors that are profoundly difficult to treat make them an attractive area of study. Future work to better understand the CSC-specific molecular pathways will be critical in understanding the mechanisms of tumorigenesis, metastasis, and treatment failures with the ultimate goal of developing novel CSC diagnostics and therapeutic targets.

## Conflict of Interest Statement

The authors declare that the research was conducted in the absence of any commercial or financial relationships that could be construed as a potential conflict of interest.
